# Fabrication and properties of interweaved poly(ether ether ketone) composite scaffolds

**DOI:** 10.1038/s41598-022-26736-4

**Published:** 2022-12-23

**Authors:** Xiaohui Song, Dengwen Shi, Wenqiang Li, Huadong Qin, Xingguo Han

**Affiliations:** 1 School of Electromechanical Engineering, Guilin University of Aerospace Technology, Guilin, 541004 China; 2 Byd Precicion Manufacture Corporation Limited, Shenzhen, 518000 China; 3 Department of Orthopaedics, Union Hospital, Tongji Medical College, Huazhong University of Science and Technology, Wuhan, 430022 China

**Keywords:** Engineering, Materials science

## Abstract

This paper interweaved scaffolds with poly(ether ether ketone) (PEEK) and poly(lactic acid)/Walnut shell/hydroxypatite (PLA/WS/HA) composites by using fused filament fabrication technology, although there was a huge difference in thermal property term between PLA and PEEK. In order to keep mechanical properties of PEEK scaffold and remedy the stress loss produced by pores, PLA/WS/HA composites were used to fill the pores with gradient form outside-in (0.4-0.8 mm, 0.6-1.0 mm, 0.8-1.2 mm and 1.6-2.0 mm). The thermal stability, tensile and compression properties, tensile fracture surface morphology, cytotoxicity and in vivo experiment were investigated. The results showed: the scaffolds were intact without any flashes and surface destruction, and kept a well thermal stability. Compared with the PEEK porous scaffolds, the tensile fracture stress and strain, compression yield stress and strain of interweaved scaffolds were dramatically enhanced by 24.1%, 438%, 359.1% and 921.2%, respectively, and they climbed to the climax at 8 wt% of WS. In vivo experiment showed that the degradation of PLA/WS/HA composites synchronized with the adhesion, proliferation and ingrowth of bone cells, keeping the stable biomechanical properties of interweaved scaffolds. Those experiments showed that interweaved PEEK-PLA/WS/HA scaffolds had the potential to be used as bone implant in tissue engineering.

## Introduction

In the field of tissue engineering (TE), current materials used as bone scaffolds mainly include titanium, ceramics and poly(ether ether ketone) (PEEK)^1^. Due to the high brittleness of ceramics and the stress shielding issue of Ti and Ti alloys, PEEK has been selected as an alternative implant for its favorable biocompatibility and good mechanical properties^2,3^. Its elastic modulus (3–4 GPa) is closer to that of cortical compact bone (14 GPa), which reduced or removed the stress shielding and bone resorption after implantation^1^. It is also radiolucent resulting in the application of radiographic evaluation^4^. PEEK has been utilized successfully in some fields, including cranioplasty, dental implants, interbody fusion, joint replacements, soft-tissue repairs, and cardiac surgery^5^.

However, PEEK is bioinert and hydrophobic^6^, whose morphology showed no obvious change after being immersed in SBF for 28 days^7^. Calcium phosphate biomaterials were often used to create bioactivity by composing with PEEK, such as (β-TCP) and hydroxyapatite (HA)^6^. Yu et al.^7^ observed that the apatite was formed on the surface of PEEK by inputting HA, indicating that the PEEK-HA composites possessed better bioactivity and hydrophilia than PEEK. PEEK/β-TCP embodied the highest alkaline phosphatase activity within 14 culture days^8^, compared with PEEK, PEEK/HA and Ti_6_Al_4_V. Therefore, for improving the bioactivity of PEEK, HA was being considered in this study.

In order to make PEEK to quite fit the structural requirement of human bone, porosity and interconnect pores should be designed and fabricated according to the structure of human bone^9^. Human bone like a sandwich, showing a functional gradient pores from the outside cortical bone to inner cancellous bone^10^. Cortical bone has dense structure with micro pores; cancellous or trabecular bone is a network structure with large pores and a low elastic modulus about 1 GPa^11^. Therefore, the structure of scaffold should be designed to cater gradient pore feature of human bones. Leong et al.^12^ and Miao et al.^10^ reviewed the characteristics, advantages and manufacturing of gradient porous biomaterials. Bittner et al.^13^ discussed the fabrication of multilayered scaffold with various AM technologies. Various materials were adopted to fabricate gradient scaffolds, including polycaprolactone (PCL)^14^, PCL-HA^15,16^ and poly(ethylene glycol)-terephthalate–poly(butylene terephthalate) (PEGT/PBT)^17^. In vitro experiment, the gradient scaffold promoted the anisotropic cell distribution more than the uniform scaffold^15^.

For manufacturing porous PEEK, several techniques have been successfully utilized, such as particulate leaching^18^, compression sintering^19^, micromachining, cold compression^20^, pressureless sintering^7^. With these methods, scaffolds with open and interconnected micro pores were obtained, providing tunnel for human tissue and enhancing dramatically the bone regeneration. However, micromachining easily caused the degradation of polymers^21^; others lacked feasibility and controllability, leading to the performance defects of implants after implantation^22^.

Additive manufacturing is able to fabricate parts with complex shapes and features, which is especially suitable to customize human bones. There are two main kinds of methods for manufacturing PEEK scaffolds, including Selective Laser Sintering (SLS)^23^ and Fused Filament Fabrication (FFF) technology (also called 3D printing). The preheating is essential for keeping the dimensional stability of the SLS parts^24^,which would age the support powders and further compromised mechanical properties^25^. The details of the impact of SLS on PEEK could be referred to literatures^23,26^. Due to the limitations of SLS of PEEK, FFF technology was focused by researchers.

Haleem et al.^27^ and Knaus et al.^2^ pointed out that FFF was the mostly preferred for printing PEEK parts, which could accurately match the patients’ anatomy. Honigmann et al.^28^ reviewed the application of 3D printed PEEK on the surgical implants. Zhang et al.^29^ used FFF to manufacture PEEK costal cartilage prosthesis. Elhattab et al.^30^ investigated properties of 3D printed PEEK scaffolds with uniform macropores (800–1800 µm). All of these researches have made excellent contributions to the development of the PEEK porous scaffold.

However, porous scaffolds with either uniform or gradient pores would sacrifice some mechanical properties. For solving this issue, several literatures provided meaningful ideas. Liu et al.^31^ fabricated scaffolds via two nozzles with two kinds of materials, one nozzle extruded poly(a-hydroxy acid) and the other was applied to tricalcium phosphate solution. These two materials can support each other. Yang et al.^32^ adopted a water-solution material as the supporter to 3D print multi-scale gradient porous scaffolds. Therefore, incorporated another biomaterial into the gradient pores of PEEK during the FFF and then removed it after implantation to make the scaffold to meet all the requirements of a tissue implant.

Poly(lactic acid) (PLA), as a kind of biodegradable, recyclable, and compostable thermoplastic^33^, has been extensively used as feedstock for FFF^34,35^ and been considered as an ideal biomaterial for TE^36^. However, like PEEK, PLA is hydrophobic^37^. And Walnut Shell (WS), a kind of natural fiber, has been authorized by Food and Drug Administration (FDA) as reducing the risk of heart disease^38^. Therefore, our research team has developed some research of FFF PLA/WS/HA composites^39^.

In this paper, the PEEK-PLA/WS/HA composite scaffolds were interweaved by using a high temperature double nozzle FFF machine. The two nozzles extruded PEEK filament and PLA/WS/HA filament, respectively. PLA/WS/HA composite was functioned as filler for filling the gradient pores of PEEK scaffolds. The effect of the pore size on the compressive and tensile properties was investigated. The microstructure of the interface between PEEK and PLA/WS/HA was analyzed. The tensile fracture surface of PEEK-PLA/WS/HA composite was characterized. The in vivo experiment was carried out to verify the effectiveness of scaffolds.

## Materials and methods

### Materials

PEEK filament was obtained as a medical-grade polymer from Henan Creatbot, China, which possesses a density of 1.3 g/cm^3^, a glass-transition temperature of 143 °C and a melting temperature of 343 °C. Poly(lactic acid) (PLA), being purchased from Nature Works LLC, USA, was a medical-grade powder material with a specific gravity of 1.24 g/cm^3^ and a melting point of 152 °C. While the Walnut Shell (WS) was gotten from a local planter in north of Guangxi, China, in a form of broken shell. Prior to use, the WS was cleaned, dried, milled and seized to average diameter of 50 μm. HA particle, being purchased from Zhejiang Emperor nano material Co., Ltd, is used as surgical implant. For improving the interfacial compatibility between particles and PLA, HA and WS were modified with alkali and silane^39^.

### Design and FEA of nozzle system

In this study, the material PEEK is a kind of high temperature polymer with a melting point of 343 °C, whereas the polymer based composite PLA/WS/HA possesses a melting temperature about 152 °C. The large temperature gap (191 °C) would cause the heat transfer from the higher nozzle system to the lower one, and hamper the smooth feeding and extrusion of the filament.

**Figure 1:**
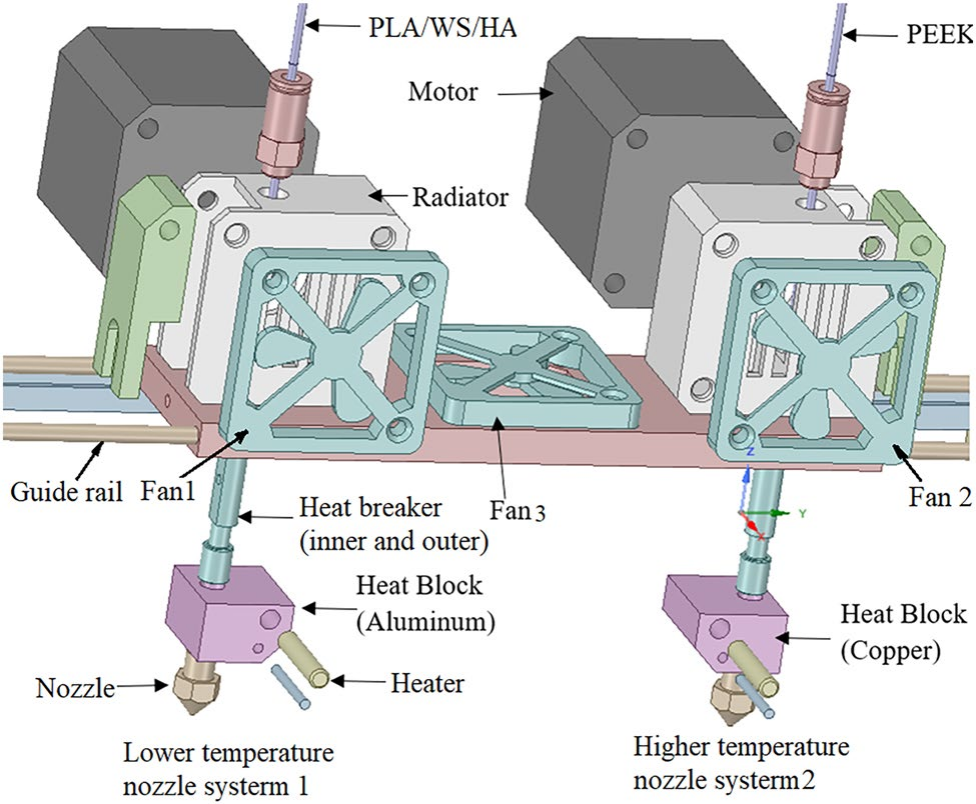
Mechanical structure of the designed nozzle system.

In order to solve this problem, a nozzle system was designed and shown in Fig. 1, which included a lower temperature nozzle system (on the left, system 1) and a higher one (on the right, system 2). The system 1 and 2 contributed to the PLA/WS/HA composite filament and the PEEK filament, respectively. There were three fans in the system: fan 1 and fan 2 were designed to cool two filament feeding mechanisms, fan 3 was designed to take away the heat between two nozzles, and to prohibit the heat transferring from the nozzle 2 to nozzle 1.

Finite element analysis (FEA) was carried out to evaluate the effectiveness of the modified dual-nozzle system. The heat transfer between two systems included: thermal conduction between contacting parts (*Q*_*1*_), convection between air and parts (*Q*_*2*_), and radiation between high-temperature and low-temperature components (*Q*_*3*_). The calculating equations of these three kinds of heat were below as^40^:

1 $$Q_{1} = - kS\frac{dT}{{dx}}$$

2 $$Q_{2} = hA\Delta T$$

3 $$Q_{3} = \varepsilon \sigma A_{1} F_{12} \left( {T_{1}^{4} - T_{2}^{4} } \right)$$

where $k$ denotes thermal conductivity (W/mK); S is the cross-sectional area of thermal conduction (${\text{m}}^{2})$; $\frac{dT}{dx}$ denotes the differential of temperature difference with heat transfer distance; $A$ denotes the surface area of parts (${\text{m}}^{2})$; $h$ is the convective heat transfer coefficient (W/m^2^K), which was determined as 60 W/m^2^ K considering the function of three fans; $\Delta T$ denotes the temperature difference between air and parts (°C); $\varepsilon$ is emissivity (0–1), which was chosen as 0.6 due to the convection heat existed mainly between two nozzles and two heat blocks; $\sigma$ equals to 5.67 × 10^–8^ W/(m^2^ K^4^); ${A}_{1}$ denotes the area of radiant surface 1, ${T}_{1}$ and ${T}_{2}$ denotes the temperature of radiant surface 1 and 2, respectively; ${F}_{12}$ is the shape coefficient.

The ambient temperature was set as 36 °C, and the heating temperature for nozzle 1 and 2 was chosen as 210 °C (PLA) and 420 °C (PEEK), respectively. Materials and thermal conductivity of various parts were shown in Table 1.

**Table 1:** Thermal property of different part.

Part	Material	Thermal conductivity (W/m °C)
Radiator, fan	Aluminum	144^41^
Nozzle	Copper	400^41^
PEEK	PEEK	0.29
PLA	PLA	0.13
Inner tube	polytetrafluoroethylene	0.4
Motor, heat breaker	Stainless steel	13.8^40,42^

### Preparing of PEEK-PLA/WS/HA scaffolds

The PEEK-PLA/WS/HA scaffolds were prepared by using the nozzle system of the FFF machine. Prior to the processing, an orthogonal experiment of FFF scaffold was carried out to determine the optimum processing parameters of FFF, shown in Table 2. Due to the melting point of PLA is about 152 °C, so that the supporting material was chosen as PEEK in the form of grid. In order to adhere PEEK onto the platform of FFF machine and prevent the part from moving during the processing, a carbon fiber platform was selected first. However, the result showed that the PEEK material was not able to adhere to the carbon fiber platform. After that, some solid glue was coated onto the carbon fiber platform. Whereas, it was hard to ensure the evenness and plainness of the glue, resulting in partial warpage of the PEEK scaffold. In addition, the glue was not able to be reclaimed and reused, leading to cost increase. Finally, polycarbon plate with a thickness of 2 mm was determined, which was effective for the FFF of PEEK and was reusable. The details of the platform choosing has been investigated in our previous study^26^. During the extrusion of one kind of material, the other material in the nozzle was keeping heated, which would easily lead to polymer degradation and carbonization, as well as nozzle clogging. Furthermore, the filament in the heat breaker will be soften because of a short resistence time. Therefore, an enough retraction length was set as 30 mm.

**Table 2:** Processing parameters of FFF for manufacturing PEEK-PLA/WS/HA scaffolds.

Parameter	PEEK	PLA/WS/HA
Nozzle heater temperature (°C)	410	210
Printing speed (mm/s)	20	50
Layer thickness (mm)	0.1	0.1
Platform temperature (°C)	70	70
Retraction length (mm)	30	30
Fill density (%)	100	100
Supporting	√	/
Platform material	Polycarbonate plate	

**Figure 2:**
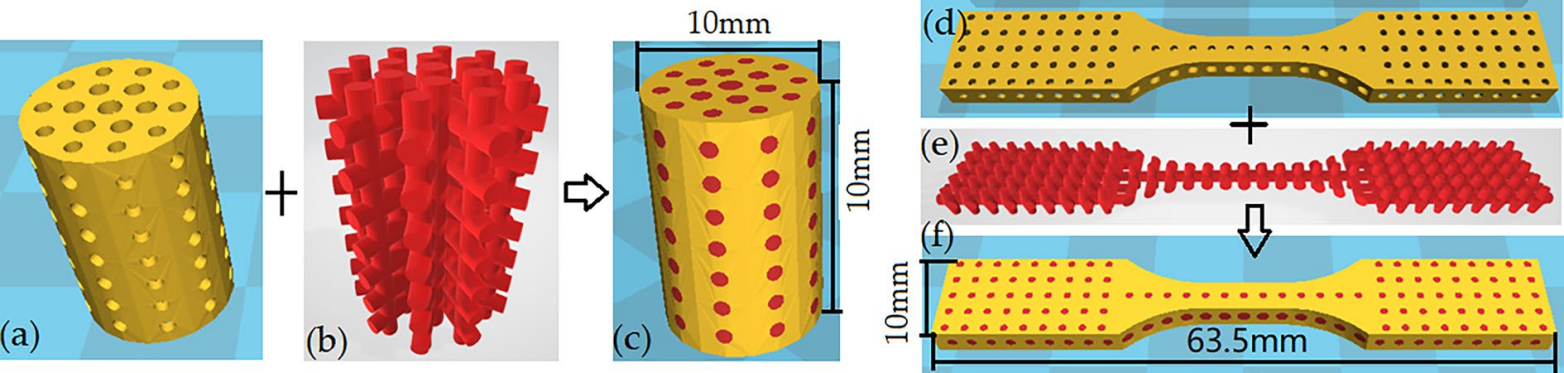
Designed model of the compressive scaffold: (**a**) PEEK, (**b**) PLA/WS/HA and (**c**) PEEK-PLA/WS/HA; and tensile scaffold: (**d**) PEEK, (**e**) PLA/WS/HA and (**f**) PEEK-PLA/WS/HA.

On the basis of the parameters in Table 2, composite scaffolds for compression and tensile testing were interwoven with PEEK and PLA/WS/HA. The models of PEEK (Fig. 2a,d) were designed as porous scaffold with gradient pores varying outside-in (0.4–0.8 mm, 0.6–1.0 mm, 0.8–1.2 mm and 1.6–2.0 mm). The PLA/WS/HA models (Fig. 2b,e) were arranged in dendritic form to fill the pores of PEEK scaffolds. In addition, two of them were integrated into PEEK-PLA/WS/HA composite scaffolds (Fig. 2c,f) with compositions shown in Table 3. Finally, cylindrical compression samples with a diameter of 10 mm and a height of 15 mm, and dog-bone-shaped tensile samples with a dimension of 63.5 mm × 9.53 mm × 3.2 mm were manufactured with PEEK-PLA/WS/HA. Furthermore, in order to estimate the cell viability of PEEK-PLA/WS/HA scaffold, implant samples with diameter of 4 mm and a height of 10 mm were fabricated with FFF for animal experiment.

**Table 3:** Compositions of PEEK-PLA/WS/HA scaffolds.

Scaffold	Pore size of gradient pore in PEEK scaffold (mm)	PLA (wt%)	WS (wt%)	HA (wt%)
S0	1.2–1.2	89	3	8
S1	0.4–0.8	89	3	8
S2	0.6–1.0	89	3	8
S3	0.8–1.2	89	3	8
S4-1	1.6–2.0	94	3	3
S4-2	1.6–2.0	89	3	8
S4-3	1.6–2.0	82	3	15
S4-4	1.6–2.0	84	8	8
S4-5	1.6–2.0	82	15	3

### Characterization

The compression and tensile testing were carried out by using a universal testing machine (Model 8800, Instron, Canton, MA). Based on ASTM 638 standard, the testing rate for tensile was set as 0.3 mm/min and the compression rate was 3 mm/min according to GBT 1041 standard. The preload for both was 0.1 N.

A simultaneous thermal analyzer (WCT-121, Beiguang Hongyuan, China) was applied to characterize the thermal stability (TG). Sample about 20–25 mg was submitted to a procedure under 20 mL/min rate of flowing nitrogen: heating to 900 °C at the rate of 10 °C/min, keeping for 5 min, and finally cooling normally.

The morphology of the tensile fracture surface was characterized. The test was carried out with a scanning electron microscopy (SEM, VEGA3 TESCAN). Prior to the test, all of the scaffolds have been gold coated for 5 min.

Bone Marrow Mesenchymal Stem Cells (BMMC) were donated from a young patient who was undergoing an orthopaedic surgery and has signed the informed consent form. The research method in this study conforms to the standard in *Declaration of* *Helsinki* and was authorized by the Ethics Review Committee of the Union Hospital affiliated to Huazhong University of Science and Technology. The MSCs were cultured in Dulbecco’s modified Eagle’s medium–F12 supplemented with 10% fetal bovine serum in humidified atmosphere at 37 °C.

The cell viability was estimated with a cell counting kit-8 (CKK-8, Dojindo, Japan). The scaffolds were placed into a 24-well plate with BMMC to inoculate cells for 24 h. Then a medium with 30 μL CCK-8 and 300 μL serum was added into each well. The cells were incubated on scaffolds at 37 °C for another 2 h. A 100 μL of cultural medium was transferred into a 96-well plate. The absorbance (OD) of each well at 450 nm was measured by using a microplate reader. The cell viability percentage (C_v_) was calculated with the formula:

4 $${\text{Cv}} \left( \% \right) = \frac{{A_{s} - A_{b} }}{{A_{0} - A_{b} }} \times 100\%$$

where A_s_ denotes absorbance in the test well, A_b_ is the absorbance in the test well without cell and scaffold, and A_0_ denotes the absorbance in the test well without scaffold.

Micro-computed tomography (micro-CT) (skyscan 1176, Bruker, America) was applied to examine the architecture of implanted samples. The testing pressure and electricity were set to 80 kV and 313 mA, respectively. Two-dimensional image slices (transverse, coronal and sagittal) were obtained, and reconstructed to three-dimensional images.

## Results and discussions

### FEA of nozzle system

**Figure 3:**
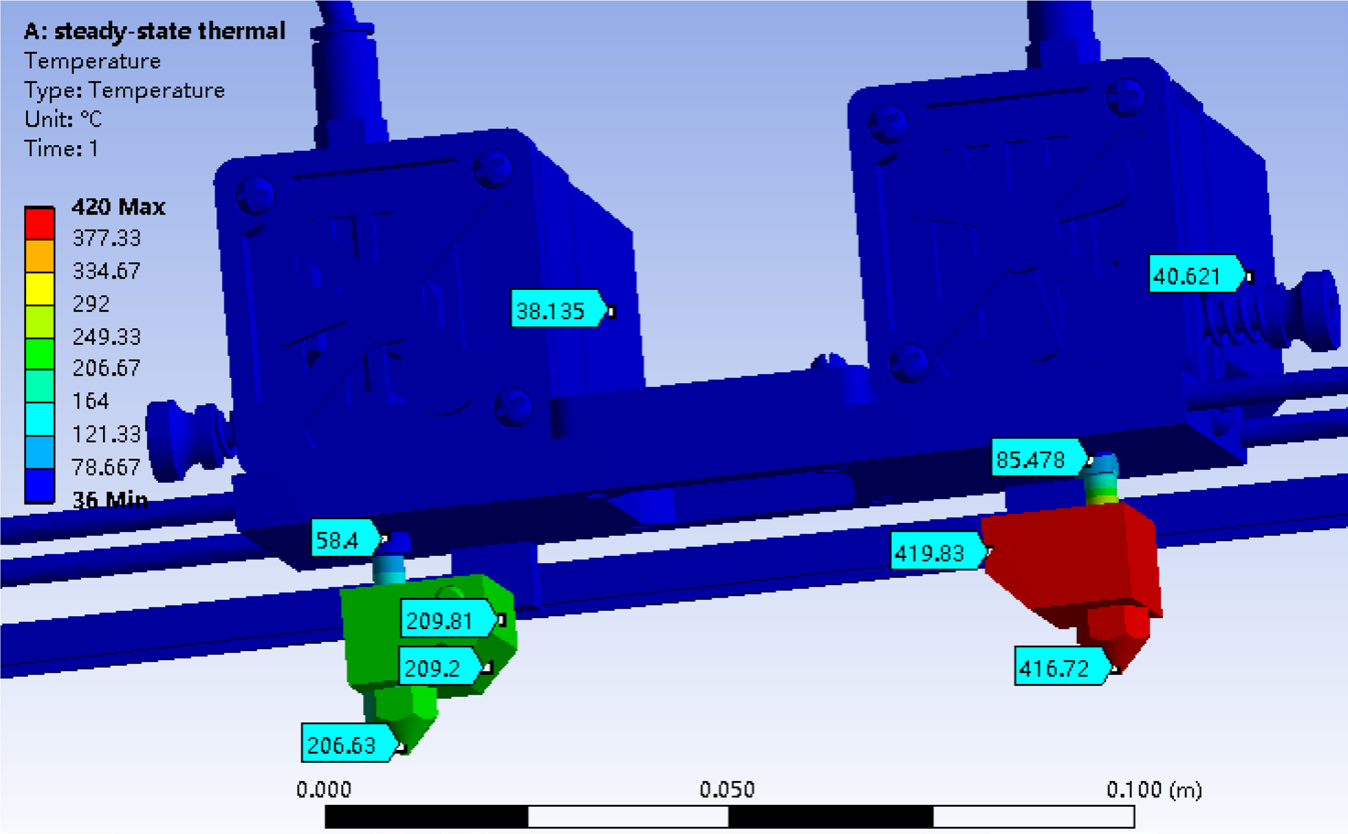
Temperature profile of the designed nozzle system.

The temperature profile of the designed nozzle system is shown in Fig. 3. The temperature of heat block 1 and nozzle 1 were 209 °C and 206 °C, respectively; the heat block 2 and nozzle 2 possessed a temperature of 419 °C and 416 °C, both were a little lower than that of their heater (210 °C and 420 °C). The results indicated that the two nozzle systems kept a stable temperature, which was very important for the stable printing and printed quality. Sun et al.^43^ proved that the safe distance between two nozzles should be wider than 16 mm. In this study, 60 mm was determined under comprehensive consideration on the assembled fan between two nozzles. The temperature above guide rail was about 38 °C, which was a safe temperature for feeding motor working and would keep filaments solid.

**Figure 4:**
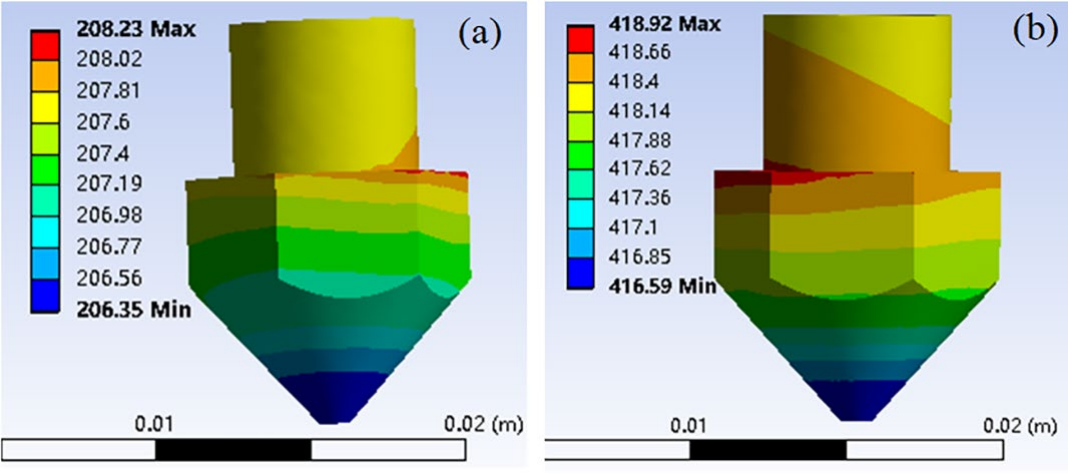
Temperature distribution of nozzle: (**a**) lower system and (**b**) higher system.

The temperature distribution of nozzles is shown in Fig. 4, which was in the range of 206.35–208.23 °C and 416.59–418.92 °C for lower and higher temperature nozzle 1 and 2, respectively. Given the lowest temperature at the outlet of nozzles, the printing temperature should be higher 2–4 °C than the setting values.

**Figure 5:**
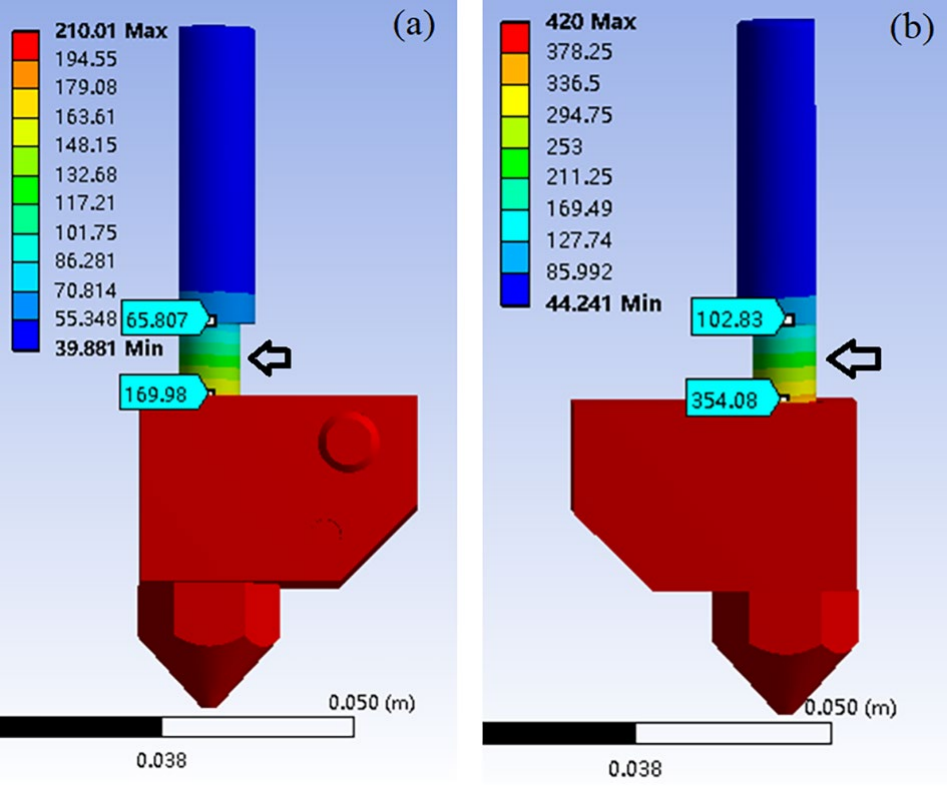
Temperature distribution of heat breaker: (**a**) lower system and (**b**) higher system.

The temperature gradient of heat breaker was dramatically wide (Fig. 5), especially on the neck area, where the temperature decreased from 354 °C (near heat block) to 102 °C. The heat breaker possessed a small diameter, leading to a weak thermal conduction. The results indicated that the heat breaker took an important role in insulating heat and preventing the filament from being soften and deformed^44^.

### The procedure of the FFF of PEEK-PLA/WS/HA scaffolds

**Figure 6:**
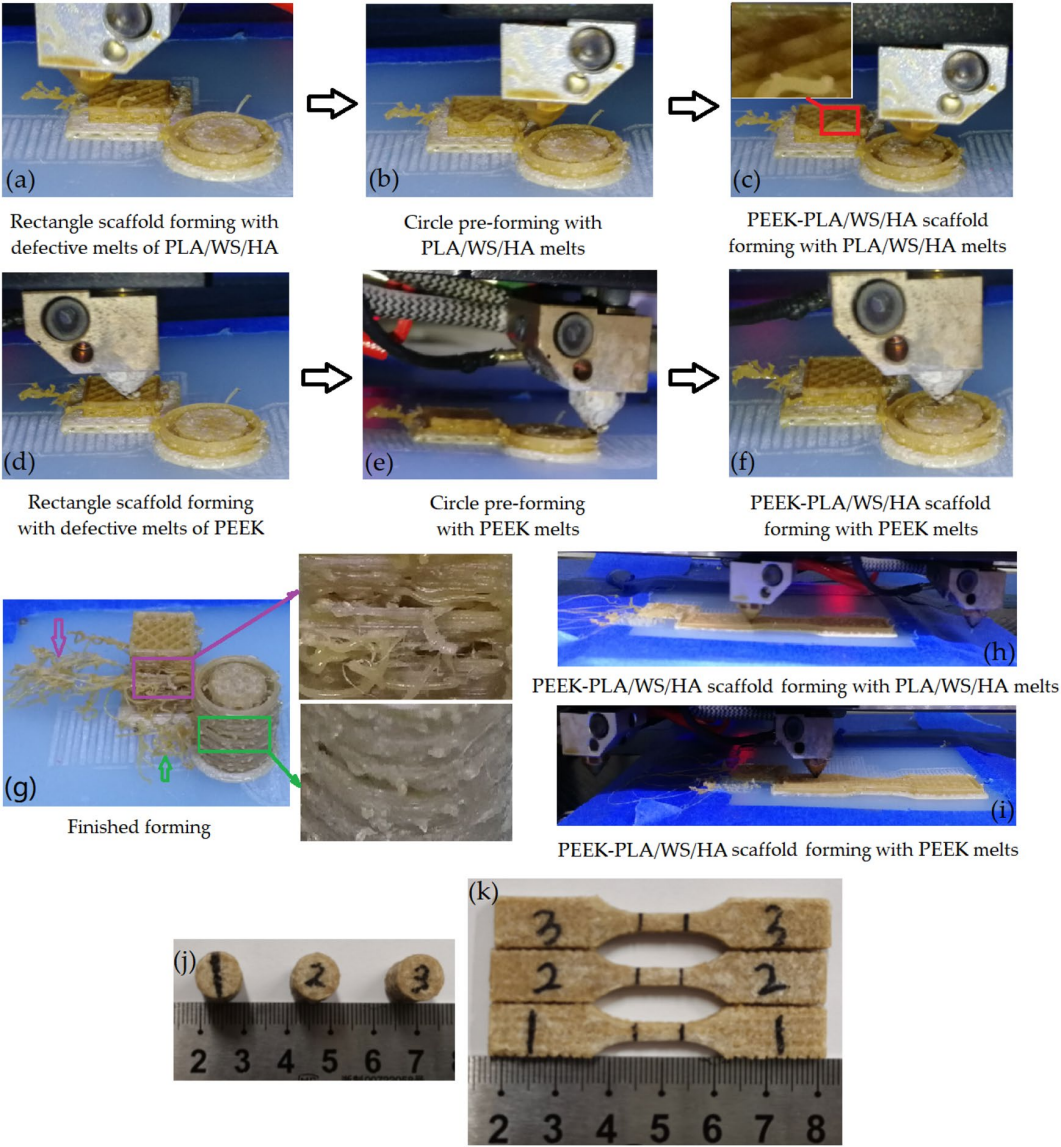
Procedure of the PEEK-PLA/WS/HA composite scaffolds.

Figure 6 shows the procedure of PEEK-PLA/WS/HA scaffolds. The processing was processed layer by layer by using two nozzles. Nozzle 1 (lower temperature nozzle) processed the composite filament of PLA/WS/HA, nozzle 2 (higher temperature nozzle) was responsible to the PEEK filament. Each of layers was carried out in three steps: (1) forming a rectangle scaffold (named residual tower) with the PEEK or PLA/WS/HA residual melts (Fig. 6a,d), (2) pre-forming a circular ring with the PEEK or PLA/WS/HA melts (Fig. 6b,e) and (3) forming the PEEK-PLA/WS/HA scaffold (Fig. 6c,f show the processing of compressive scaffold; Fig. 6h,i give the processing of tensile scaffold.).

Although an enough retraction length has been set, the residual melts in the nozzle couldn’t be retracted. Therefore, those residual melts should be extruded before fabricating the part. It can be seen in Fig. 6c (the red rectangle), the melts has degraded severely as the color turned into brown. A little extruded polymer flied obviously out of the residual tower (purple arrow), resulting in the surface destruction of the tower (purple rectangle). During the waiting time, the melt in the nozzle absorbed too much energy, resulting in the decrease of its viscosity and the increase of its flowability. Therefore, the extrudate was out of control after being extruded, and severe flashed on the surface of the tower. Although the surface condition of the circular ring was much better than that of the tower, there were still a few of flashes (green arrow) and surface damage of the circle (green rectangle). After clearing the residents in the form of residual tower and circular ring, the compressive and tensile scaffolds were done and shown in Fig. 6j,k, respectively.

It can be seen that scaffolds were interweaved with PEEK and PLA/WS/HA composites, where the PEEK material played the main supporting function and gradient decreased outside-in like a sandwich human bone^10^. PLA/WS/HA composites played the role of biodegradable material. Both of the scaffolds were intact without any flashes and surface damage, indicating a well mechanical property and supporting function when being implanted as bone scaffolds. In addition, the surface of the scaffolds was rough, which was beneficial to the bone cell adhesion and osseointegration of implants^45^.

### Thermal stability of PEEK in various states

**Figure 7:**
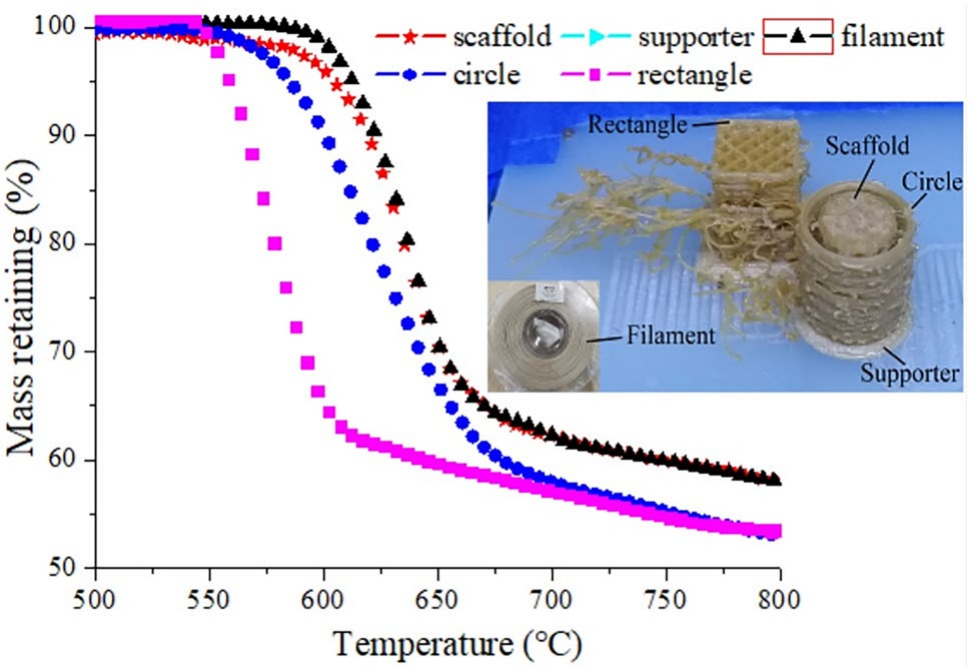
Thermal stability of PEEK in various states.

In order to further prove the effectiveness of the FFF procedure, the Thermogravimetric Analysis (TGA) of PEEK material in various states was carried out, shown in Fig. 7. It can be seen that all samples possessed one-step degradation during the whole testing procedure. The TG curve of PEEK filament was at the rightmost, showing the most thermal stability, while the PEEK rectangle displayed the poorest thermal stability with the onset degradation temperature around 550 °C. According to the printing procedure in Fig. 6, the PEEK nozzle paused after finishing one layer printing of PEEK, and then the other nozzle printed the PLA/WS/HA composite. During single layer printing, the residual PEEK melt in the PEEK nozzle was kept its printing temperature, resulting in the thermal degradation. Therefore, the PEEK rectangle, representing most of the residual PEEK melt, performed worst among all states of PEEK. A few of residual PEEK melts was printed as PEEK circle, resulting in poorer thermal stability. The PEEK scaffold began to degrade almost at a similar temperature as the PEEK supporter. Those results showed that the quality of scaffold was guaranteed by the design of PEEK rectangle and circle.

### Mechanical properties of the FFF PEEK-PLA/WS/HA scaffolds

The mechanical stability is the critical factor for the remodeling of bone tissue. The lack of mechanical loading easily leads to the bone mass reduction^46^. The PEEK scaffolds possessed interconnected pores gradient in a range of 0.4–0.8 mm, 0.6–1.0 mm, 0.8–1.2 mm and 1.6–2.0 mm outside-in. The structural details and mechanical properties of the PEEK porous scaffolds have been reported in our former published research^26^. The later differed from the former was that the pores within the former were filled with PLA/WS/HA composites and resulted in interweaved PEEK-PLA/WS/HA scaffolds. Furthermore, the larger of the pore size, the more content of the PLA/WS/HA composites. After being implanted, the PLA/WS/HA composites could improve the mechanical properties of the PEEK scaffold. Therefore, the mechanical properties of scaffolds interweaved by PEEK and PLA/WS/HA composite were investigated.

**Figure 8:**
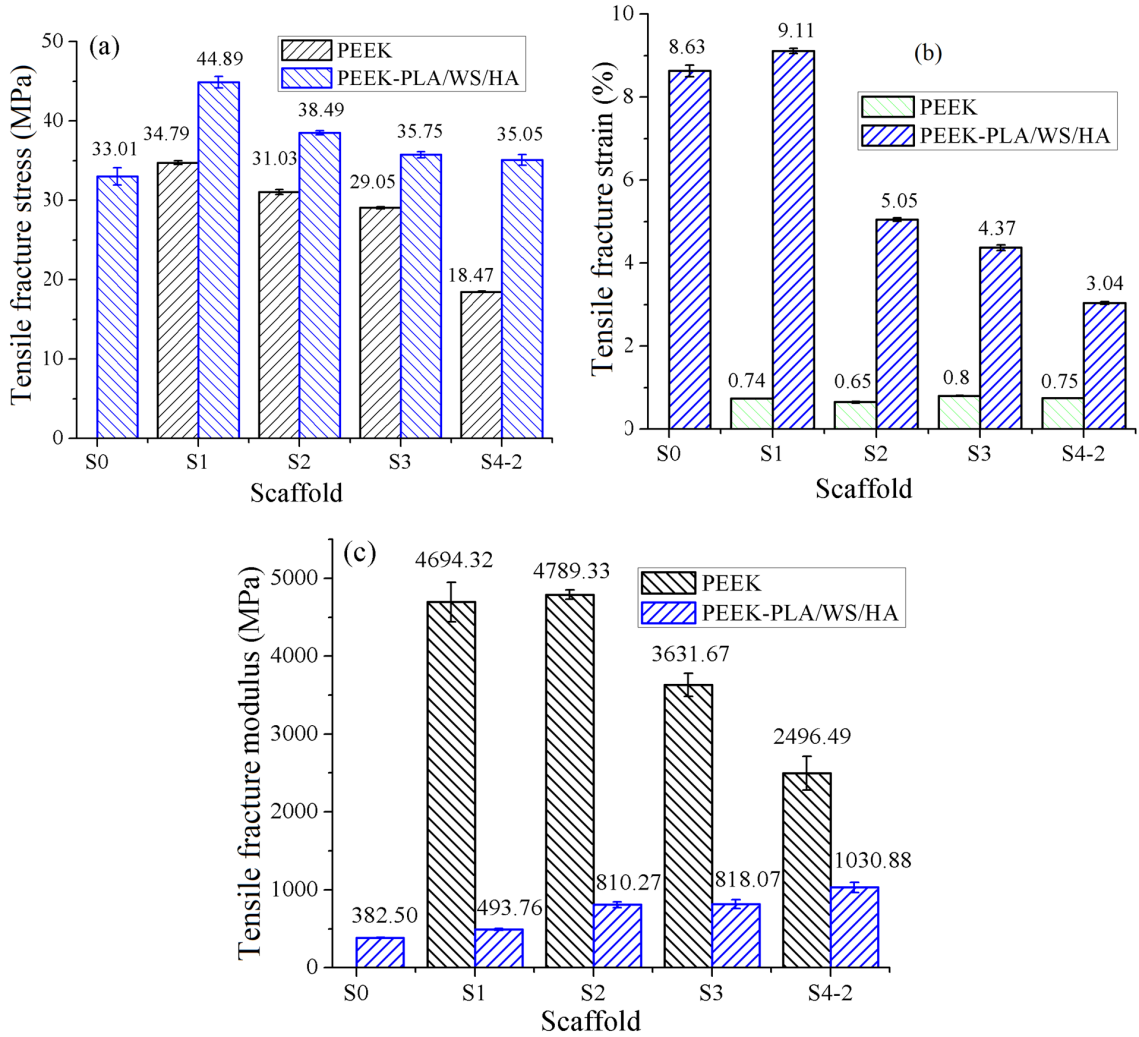
Tensile fracture properties of FFF PEEK scaffolds^26^ and PEEK-PLA/WS/HA scaffolds: (**a**) stress, (**b**) strain and (**c**) modulus.

Figure 8 gives the ultimate tensile properties of FFF PEEK and interweaved PEEK-PLA/WS/HA scaffolds. It was obvious from the fracture stress term (Fig. 8a), that the gradient interweaved scaffolds possessed enhanced fracture stress (more than 35.29 MPa), when compared to PEEK scaffolds with uniform pores (S0, 33.01 MPa). When speaking of scaffolds with gradient pore, PEEK-PLA/WS/HA performed much better than PEEK; S4-2 got the highest increment from 18.47 to 35.29 MPa; others increased about 7-10 MPa.

**Figure 9:**
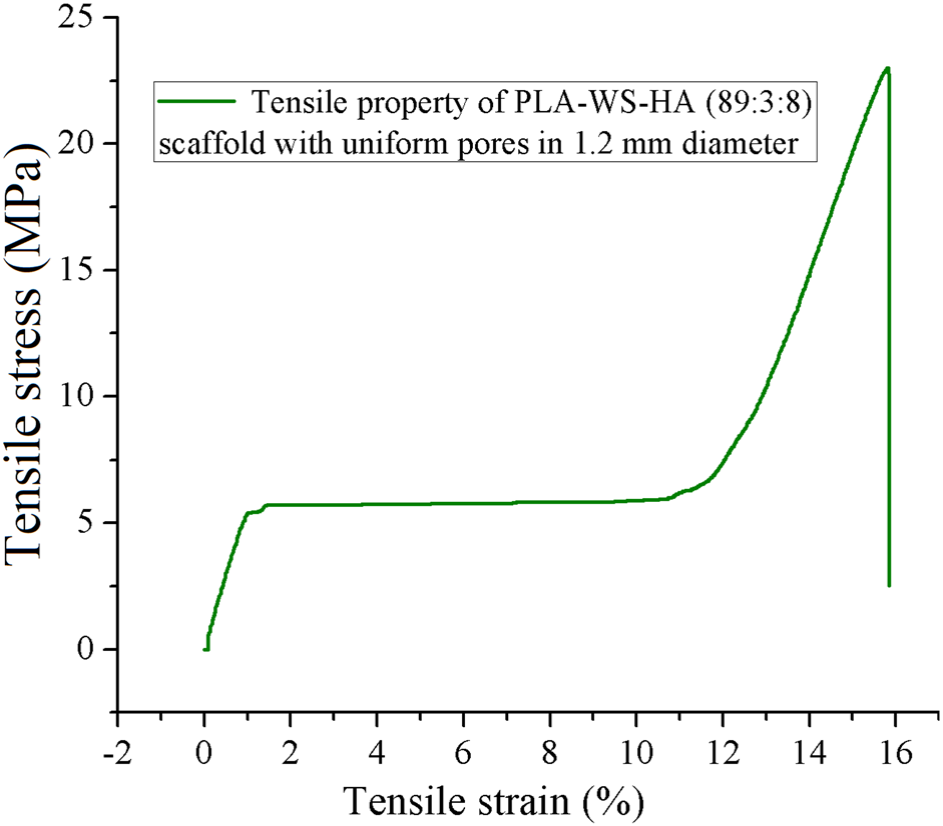
Tensile properties of FFF PLA-WS-HA scaffold.

The tensile fracture strain of PEEK gradient scaffolds was less than 1%, embodying a very weak toughness. Fortunately, the fracture strain of interweaved scaffolds rocketed dramatically to 9.11% (S1) with an increment in range of 438% (S4-2)-1131% (S1), manifesting an excellent toughness. This result indicated that the fracture strain was enhanced by incorporating PLA-WS-HA composites. Figure 9 shows the function of ultimate tensile fracture stress and fracture strain of PLA-WS-HA scaffold with uniform pores in 1.2 mm diameter. It can be seen that the PLA-WS-HA scaffold possessed only 22.95 MPa of ultimate tensile fracture stress, but an excellent fracture strain of 15.85%. The PLA-WS-HA scaffold also had a long yield plateau, performing a perfect buffer function, which further promoted the strain of interweaved scaffolds.

Figure 8c compares the tensile fracture modulus of PEEK scaffolds with interweaved PEEK-PLA/WS/HA scaffolds. It can be seen that the modulus of PEEK was much higher than that of interweaved scaffolds, indicating that the later possessed smaller stiffness. This result was contributed to the incorporation of PLA composites, whose tensile fracture modulus was 144.79 MPa and much smaller than that of PEEK. The PLA-WS-HA composite was more flexible than PEEK, leading to a decrease in the stiffness of PEEK.

**Figure 10:**
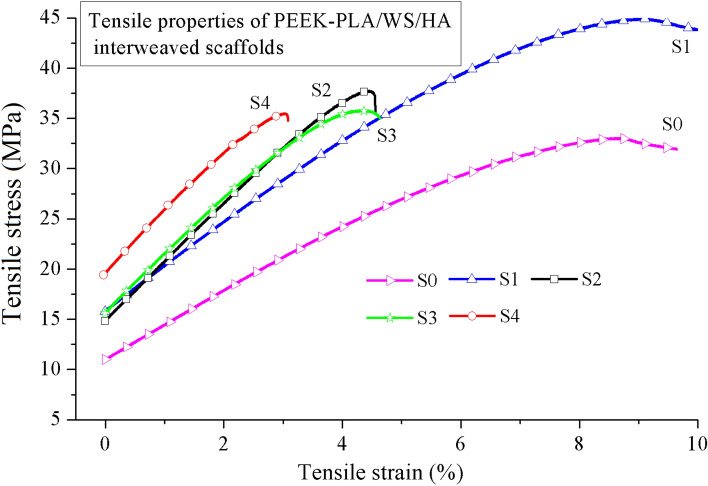
Tensile properties of FFF PEEK-PLA/WS/HA scaffold.

The relationship between tensile stress and strain of interweaved scaffolds is shown in Fig. 10. With the increase of PLA/WS/HA content, the tensile fracture stress and fracture strain of interweaved scaffolds decreased from 44.89 MPa and 9.11% to 35.29 MPa and 3.03%, respectively, which can be contributed to the weaker fracture stress of PLA/WS/HA scaffold (22.95 MPa). During the FFF processing, the contained WS and HA in PLA had totally different nature to PEEK, resulting in micro pores or defects when contacting^47^. Although the PLA/WS/HA scaffold had excellent tensile fracture strain, it did not neutralize the effect of porous PEEK, which possessed less than 1% of tensile fracture strain. Therefore, the tensile fracture strain of interweaved scaffolds did not increase with the rise of PLA/WS/HA weight ratio. This result also led to the modulus increase of interweaved scaffolds, indicating the enhancement of stiffness.

**Figure 11:**
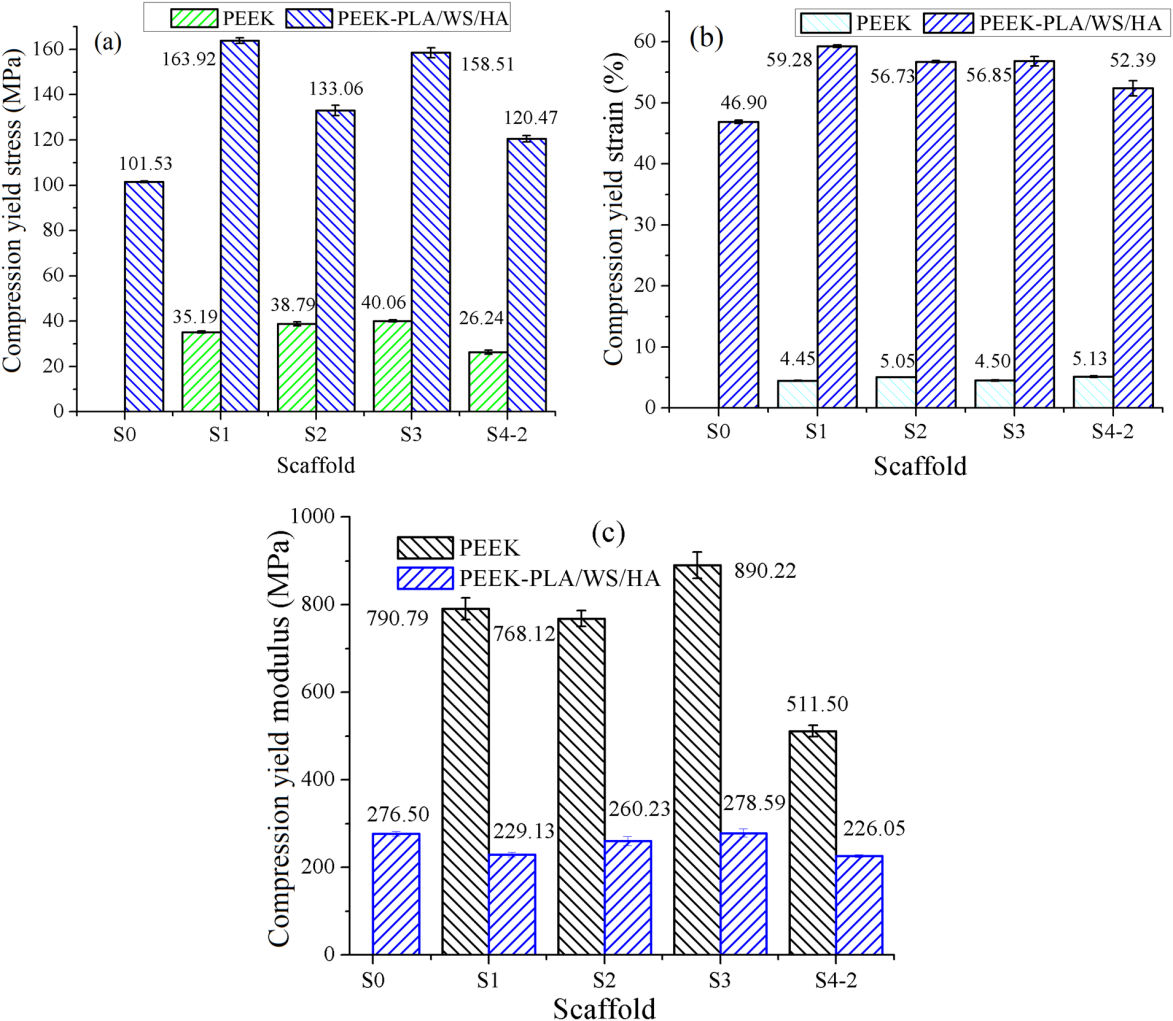
Compression yield properties of FFF PEEK scaffolds^26^ and PEEK-PLA/WS/HA scaffolds: (**a**) stress, (**b**) strain and (**c**) modulus.

**Figure 12:**
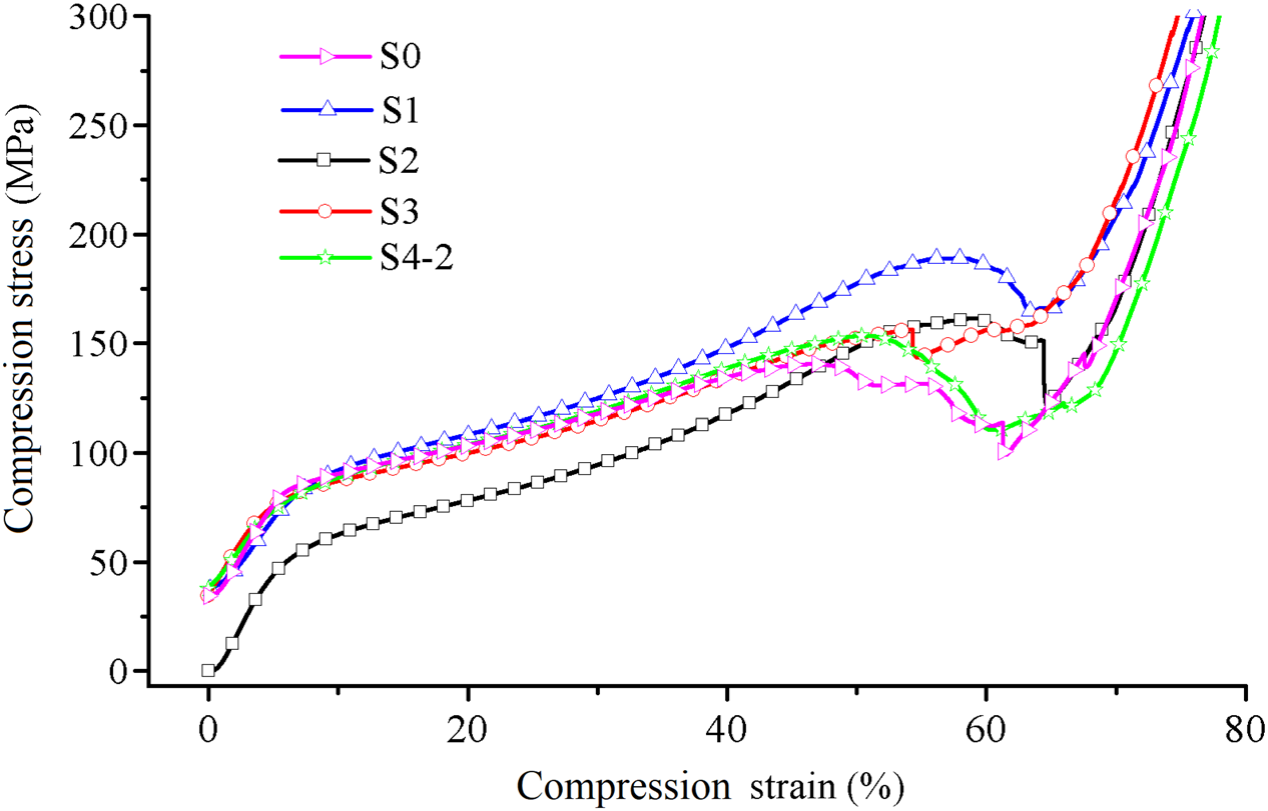
Compression properties of FFF PEEK-PLA/WS/HA scaffolds.

Figure 11 demonstrates the compression yield properties of PEEK scaffold and PEEK/PLA-WS-HA scaffold. The compression properties of PLA-WS-HA scaffold were reported in our earlier study^48^. Although the compression yield stress and yield strain of PEEK scaffold and PLA-WS-HA scaffold were only 26.24–40.04 MPa and 4.45–5.13%, 32.15 MPa and 6.35%^48^, respectively, the yield stress and yield strain of PEEK/PLA-WS-HA scaffold were enhanced dramatically to 101.53–163.92 MPa and 46.9–59.28% for each, indicating a brilliant mechanical stability when being implanted. Figures 11 and 12 show that S1 and S4-2 scaffolds performed the maximum and minimum increment by 128.73 MPa and 94.23 MPa in stress, and 54.83% and 47.23% in strain. This result contributed to the PLA/WS/HA composites filling pores of PEEK, thus an interweaved scaffold was formed. The two materials PEEK and PLA/WS/HA supported each other during the compression procedure, resulting in the improvement of compression yield stress and yield strain.

On the contrary, the compression modulus of interweaved scaffolds plummeted violently from 511.5–890.22 MPa to 226.05–278.59 MPa. This result might due to the more flexible material PLA/WS/HA strengthed the toughness of interweaved scaffold but reduced the stiffness.

Those results indicated that the mechanical stress and strain of porous scaffolds were enhanced by incorporating another material to fill the pore. Other literatures also got the similar results. Liu et al.^31^ used double nozzles to print collagen and PLGA, and concluded that composite scaffold enjoyed the sound mechanical properties of two materials. The improvement in mechanical properties of scaffolds improved the mechanical stability after being implanted in human body, where PLA/WS/HA composites and PEEK gradient scaffold supported each other. The PLA/WS/HA composites would biodegrade gradually and provided channels for bone cells adhesion, diffusion, penetration and proliferation. Therefore, interweaved scaffolds are more suitable for implantation than PEEK scaffolds.

### Effect of HA and WS ratio on mechanical properties of the FFF PEEK-PLA/WS/HA scaffolds

**Figure 13:**
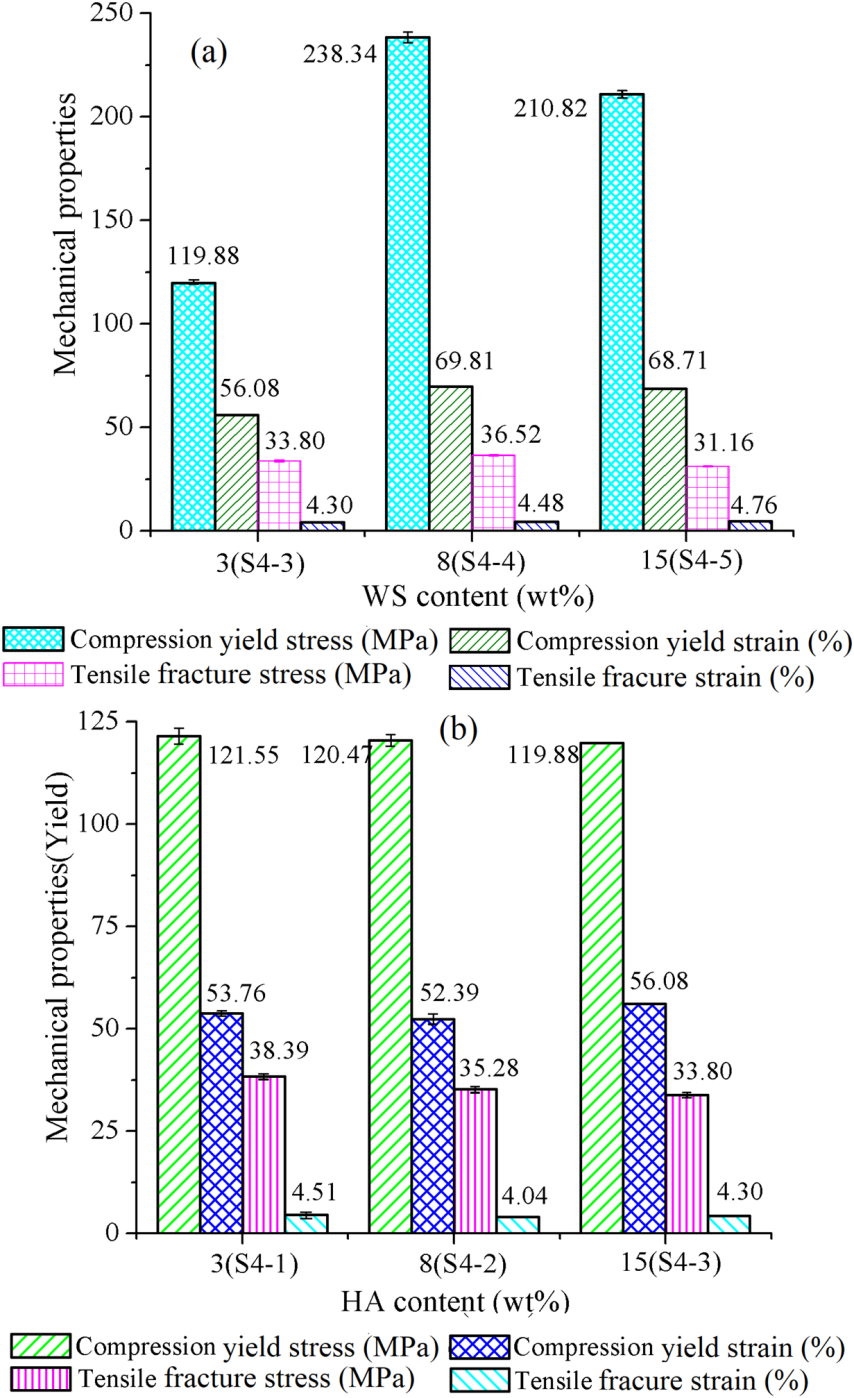
Mechanical properties of FFF PEEK-PLA/WS/HA scaffolds under content effect of: (**a**) WS and (**b**) HA.

Walnut shell (WS) is a kind of biomedical material, which reduced the risk of heart disease^38^. Figure 13a shows that the content of WS had an obvious effect on mechanical properties of interweaved PEEK-PLA/WS/HA scaffolds. Among the three scaffolds, the scaffold S4-2 (8 wt% WS) was the best, and its compression yield stress, strain and tensile fracture stress were 238.34 MPa, 69.81% and 36.52 MPa, respectively. The scaffold S4-5 (15 wt% WS) ranked the second in terms of compression yield stress and tensile fracture strain. Therefore, 8 wt% of WS was the optimum choice for scaffolds, which was in accordance with the study on PLA-WS-HA^48^. Liu et al.^49^ and Song et al.^50^ also proved that 10 wt% of walnut performed best in tensile fracture stress when compared to other ratios. They found that WS accelerated the degradation of PLA, which hence provides room for cells ingrowth. Further increasing the ratio of WS aggravated the aggregation of WS in PLA matrix, leading to poor interfacial compatibility and dropped fracture stress.

Figure 13b shows that increasing HA content (3-15 wt%) reduced moderately the mechanical stress of interweaved scaffolds, although the effect of HA was not obvious. Literature^48^ proved that HA particles distributed uniformly in PLA matrix, resulting in the stress level keeping. The compression yield stress was kept around 120 MPa, and the compression yield strain, tensile fracture stress and tensile fracture strain varied between 52% and 56.1%, 33-39 MPa and 4-4.51%, respectively. The existence of HA in scaffold enhanced the osteogenesis, diffusion and proliferation of bone cells^51^ and reduced the accumulation rate of defects^52^. Therefore, given the little effect of HA content on the mechanical properties, 15 wt% content of HA was the optimum choice in this paper.

### The morphology of FFF PEEK-PLA/WS/HA scaffolds

**Figure 14:**
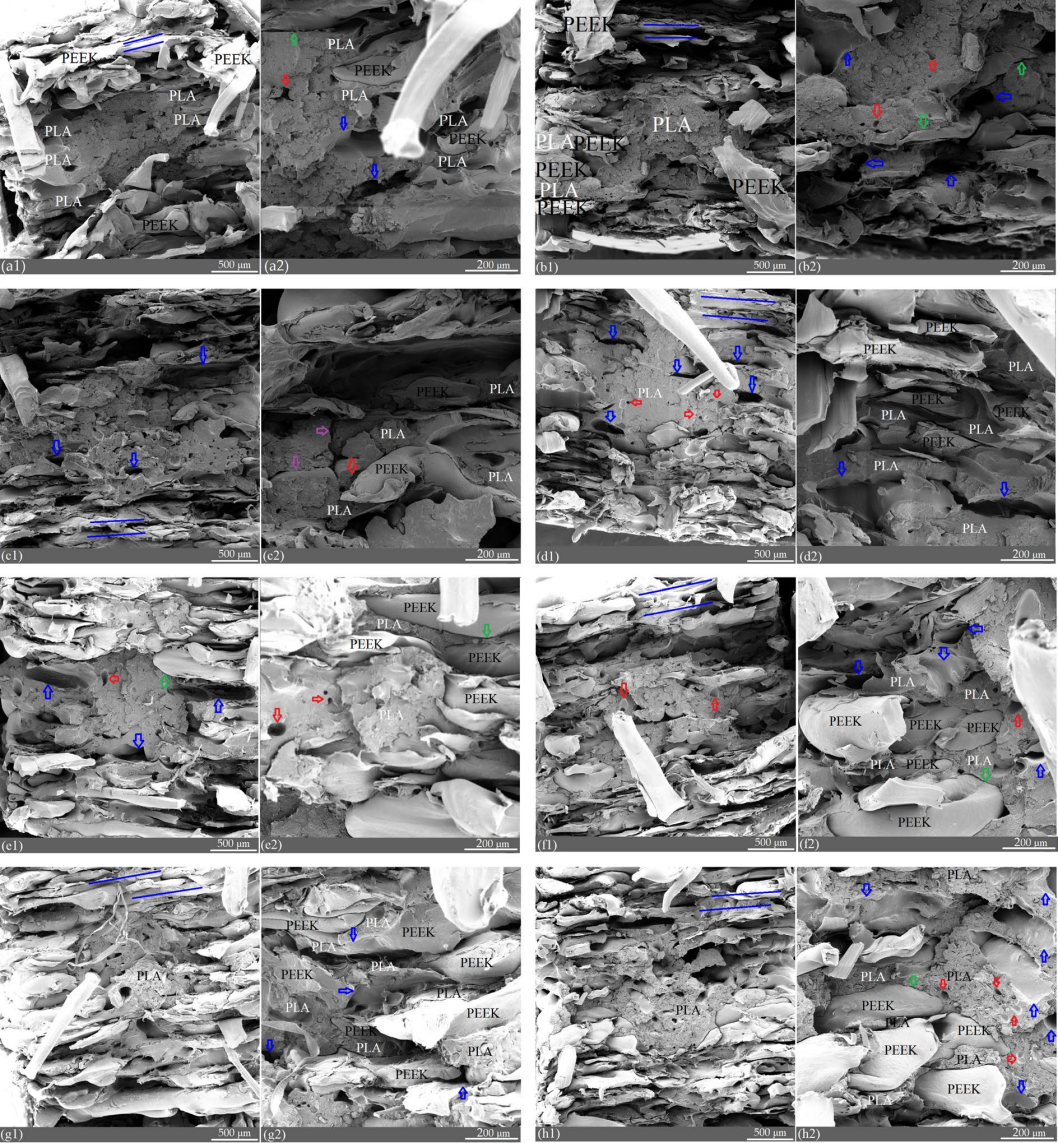
Tensile fracture morphology of PEEK-PLA/WA/HA scaffolds: (**a1**)–(**h1**), S1, S2, S3, S4-1, S4-2, S4-3, S4-4, S4-5; (**a2**)–(**h2**), the enlarged figure of (a1)-(h1), respectively.

Figure 14 shows the tensile fracture surface morphology of the PEEK-PLA/WS/HA scaffolds prepared by FFF technology. All the fractural surfaces showed interweaved pure PEEK and PLA/WS/HA composites. Along the printed line (blue line, Fig 14a1, b1, c1, d1, e1, f1 and h1), and from outside to inside of the scaffold, the amount of PEEK material decreased gradient, whereas that of PLA/WS/HA composites increased gradually. PEEK material was drew out along its printing direction (blue line) and strongly transformed, indicating excellent toughness. The surface of the PLA/WS/HA composites was relatively smooth, indicating a brittle fracture.

There were clearance (green arrow, Fig 14a2, b2, c1, f2 and h2) and delamination between PEEK layer and PLA/WS/HA composite layer. With the increase of PLA/WS/HA composite content, the clearance gradually aggravated and evolved into delamination (Fig 14d2, e2, f2, e2 and h2), indicating poor interfacial compatibility. The delamination and clearance would hamper the force transfer during tensile process. This result can explain why the tensile fracture stress and fracture strain reduced with the rise of PLA/WS/HA ratio.

There was crack in the PLA/WS/HA composite interface (Fig. 14c2, purple arrow), showing poor bearing capacity. FFF is a kind of rapid melting and rapid freezing processing^53^. Firstly, the Lifshitz-van der Waals surface tension of PLA was determined as 41.6 J/mm^2^^54^, whereas that of PEEK was tested as 3.92 * 10^−3^ J/mm^2^^55^. Therefore, the huge difference of surface tension between PEEK and PLA led to the issue of interfacial compatibility. Secondly, the crystallization temperatures of PEEK and PLA were about 315 °C^56^ and 102 °C^48^, producing different crystallization time. The melting points of PEEK and PLA were around 343 °C^26^ and 165 °C^48^, leading to the different cooling speed. The result meant that when PLA has been solidified, PEEK was still at the state of high elasticity. The good aspect was that when PLA and PEEK contacted each other, heat could be transferred from PEEK to PLA and softened PLA. Thus the vibration and Brownian motion of molecules in the surface layer was beneficial to the compatibility between PEEK and PLA. While the bad point was that different cooling speed and crystallization time led to the different orientation of molecules, resulting in the the micro pores and macro defections (delamination and crack).

Although the PLA/WS/HA composites weakened the fracture stress of interweaved scaffolds, the interlaced structure allowed them to support each other physically. This interlaced structure displayed apparently in Fig. 14d2, e2, f2, e2 and h2, which would strengthen the scaffold on one hand. However, the stronger PEEK was separated from the weaker PLA/WS/HA composites, which lowered the fracture stress of scaffolds on the other hand. Meanwhile, the interfacial compatibility between PLA and WS, as well as HA should be considered. It can be seen that there were pores (red arrow) in PLA/WS/HA composites, which was due to the pull-out of WS or HA particles. This phenomenon was especially obvious in sample S4-5 (Fig. 14h2), which contained 15 wt% of WS. The result contributed to the aggregation of WS and HA in PLA matrix, which weakened the interfacial compatibility between PLA and fillers^48^. The increased PLA/WS/HA content exacerbated the problem of interfacial compatibility with PEEK, resulting in the pull-out of PEEK and the reduction of tensile properties.

Fortunately, all the defection (clearance, delamination, pores and crack) will provide room or channels for osteocyte infiltration. When being implanted, PLA/WS/HA composites expose directly to the osteocyte environment, accelerating the biodegradation of PLA and promoting cells to adhesion, penetration and proliferation.

### Cell viability

**Figure 15:**
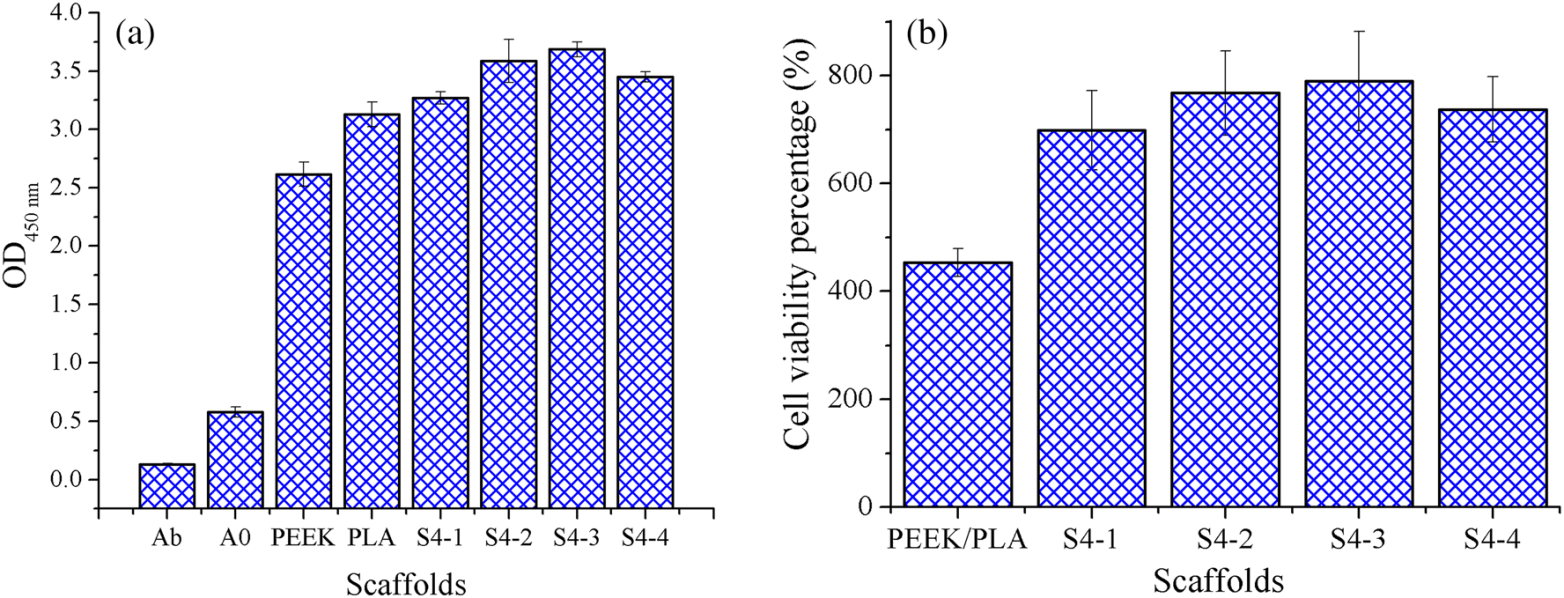
(**a**) Absorbance and (**b**) cell viability percentage of scaffolds evaluated by CCK-8 (Ab denotes the test well without cell and scaffold, and A0 denotes the test well without scaffold).

Figure 15 gives the absorbance (OD) and cell viability (C_v_) of scaffolds. All the OD values of scaffolds were dramatically enhanced when being compared with those of Blank. This result indicated that both PEEK/PLA and PEEK-PLA/WS/HA composites had no cytotoxicity and stimulated the growth of cells. PEEK-PLA/WS/HA composite scaffolds demonstrated bigger absorbance and stronger cell viability (C_v_) than PEEK and PLA scaffolds, indicating the input of WS and HA was beneficial to the cell proliferation and growth. Meanwhile, OD value and C_v_ increased steadily with the increase of HA content (3-15 wt%, from S4-1 to S4-3). PLA was an ideal biomaterial for TE^36^, WS was beneficial to the reduction of heart disease^38^ and HA was able to enhance the osteoinductivity of scaffolds^57^. Therefore, PLA/WS/HA composites not only improved the mechanical properties of PEEK, but also enhanced the cell viability.

**Figure 16:**
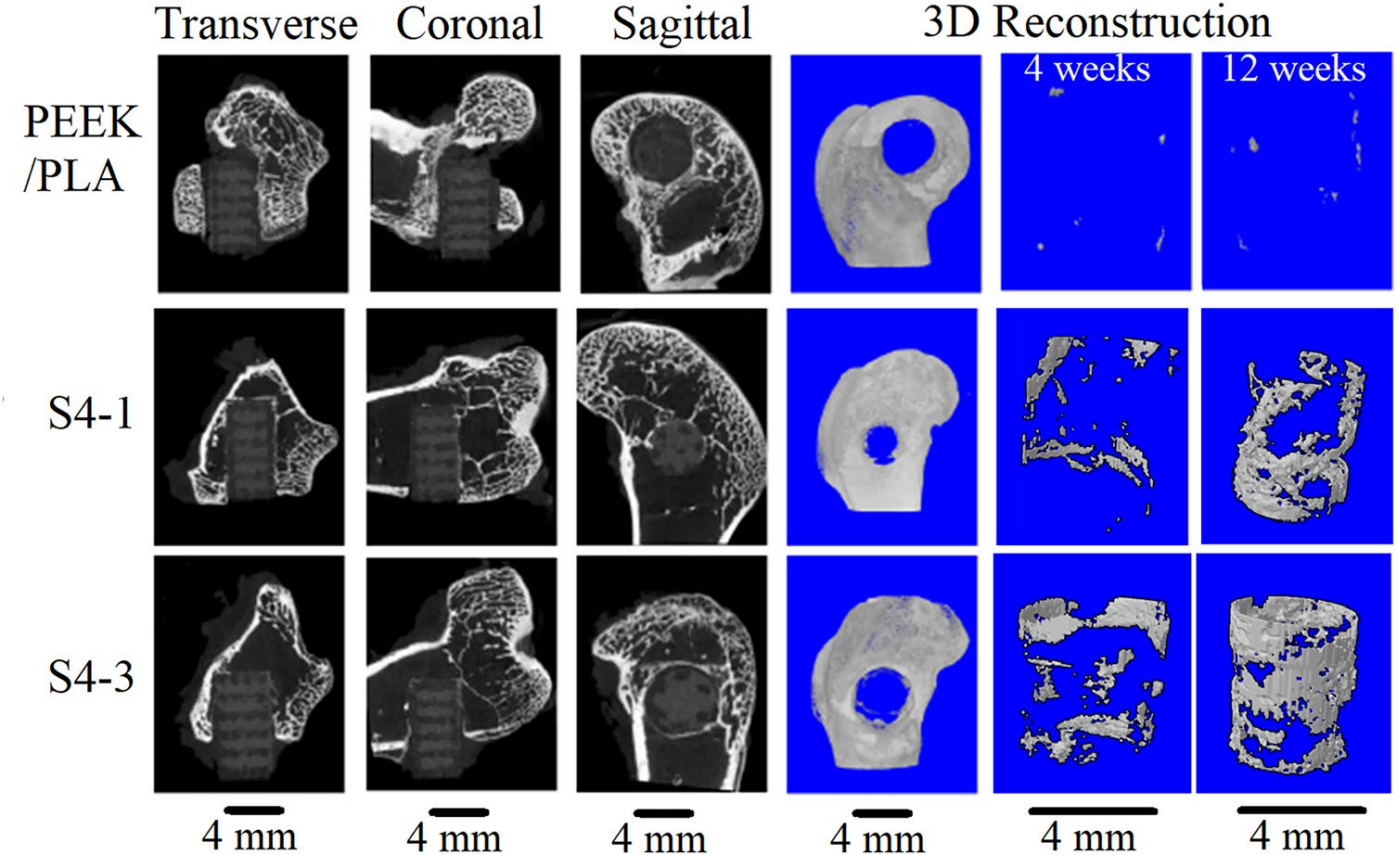
New bone formations of the PEEK/PLA and PEEK-PLA/WS/HA scaffolds as evaluated by micro-CT. 2D (transverse, coronal, and sagittal) radiographs and 3D reconstruction are shown at 4 and 12 weeks after surgery.

After being planted in distal femur of rabbits for 4 and 12 weeks respectively, the formation of new bone on different scaffolds were observed and shown in Fig. 16. With the time increasing, the number of new bone on PEEK/PLA scaffold almost remained unchanged but those on S4-1 and S4-3 scaffolds had obvious increase. Furthermore, the bone volume on S4-1 and S4-3 scaffolds was far bigger than that on PEEK/PLA scaffold, indicating that the room for bone cell ingrowth and spread was limited by the very low degradation speed of PLA^37^. The water-immersion test proved that the input of WS and HA enhanced the hydrophilia of PLA and accelerated its degradation^48^, leading to more new bone generation. Compared with S4-1, the well S4-3 performed better in terms of new bone generation, for its more content of HA (15 wt%).

## Conclusion

Although there were huge differences in thermal property and surface tension terms between Poly(lactic acid) (PLA) and Poly(ether ether ketone) (PEEK), this paper successfully interweaved scaffolds with PEEK and poly(lactic acid)/Walnut shell/Hydroxypatite (PLA/WS/HA) composites by using fused filament fabrication (FFF) technology. A double extruder system with one higher temperature nozzle and one lower was designed. The higher one was responsible for PEEK and the lower one was accountable to PLA/WS/HA composites. PLA/WS/HA composites functioned as filler to strengthen the PEEK scaffolds to keep mechanical properties and to remedy the stress loss produced by pores. PLA/WS/HA composites filled PEEK scaffolds with gradient form outside-in (0.4-0.8 mm, 0.6-1.0 mm, 0.8-1.2 mm and 1.6-2.0 mm), which conformed to the structure of human bone. The interweaved scaffolds were intact, free from flashes and surface damage, and had good thermal stability.

The mechanical tests showed: the tensile fracture stress and fracture strain, compression yield stress and yield strain of interweaved scaffolds were dramatically enhanced by 24.1%, 438%, 359.1% and 921.2%, respectively, compared with the PEEK porous scaffolds. The tensile fracture stress, compression yield stress and strain climbed to the climax (36.52 MPa, 238.34 MPa and 69.81%) at 8 wt% of WS.

In vivo and in vitro experiment showed that interweaved scaffolds was no cytotoxicity. The degradation of PLA/WS/HA composites synchronized with the adhesion, proliferation and ingrowth of bone cells, keeping the stable biomechanical properties of interweaved scaffolds. Those interweaved PEEK-PLA/WS/HA scaffolds have the potential to be used as bone implant in tissue engineering.

## Data Availability

All authors statement that “All data generated or analysed during this study are included in this published article”.
